# Acknowledgement to Reviewers 2025

**DOI:** 10.3389/phrs.2026.1609824

**Published:** 2026-04-27

**Authors:** PHR Editorial Office

**Affiliations:** Swiss School of Public Health (SSPH+), Zürich, Switzerland

The Editors of *Public Health Reviews* would like to thank all Reviewers in 2025 for the time they have spent and the valuable contributions they have made to ensure the scientific quality of the journal.

Victoria Aceti Chlebus, Canada

Noel Ackermann, Switzerland

Feyi Adepoju, Nigeria

Ugochukwu Agbakwuru, United States

Ahmed Alhadyan, Saudi Arabia

Sageda Ali, Egypt

Lola S. Almendros, Mexico

Uchechukwu Apugo, United Kingdom

Mohamad Azwan Aziz, Malaysia

Ifeoluwa Babatunde, United States

Suzanne Babich, United States

Muhammad Balogun, Nigeria

Abibata Barro, Switzerland

Helene Barroy, Switzerland

Goran Belojević, Serbia

Mandla Bhuda, South Africa

Vesna Bjegovic-Mikanovic, Serbia

Lorenzo Blandi, Italy

Marco Antonio Catussi Paschoalotto, Ecuador

Anusha Chander, Australia

Yanbing Chen, United States

Joana Costa, Portugal

Delphine Sophie Courvoisier, Switzerland

Mahmood Dalhat, Nigeria

Zuhair Dardona, Palestine

Carlo De Pietro, Switzerland

Ajay Dholakia, United States

Marcio Daniel Dias De Almeida E Silva, Portugal

Connie J. Evashwick, United States

Sunday Olutayo Fakunle, Nigeria

Marco Faytong-Haro, Portugal

Adília Fernandes, Portugal

Laura Fernandez-Bueno, Spain

Siobhan Fitzpatrick, Switzerland

Ingrid Friesema, Netherlands

Susana García, Colombia

Igor Garcia Ballhausen Sampaio, Brazil

Fifonsi Adjidossi Gbeasor-Komlanvi, Togo

Bruno Giesteira, Portugal

Isabelle Goupil-Sormany, Canada

Emily Hadley, United States

Othmane Hajji, Morocco

Dongwoon Han, Republic of Korea

Herwansyah Herwansyah, Indonesia

Francesca Horne, United Kingdom

Ismi Nurwaqiah Ibnu, Indonesia

Maria Florencia Incaurgarat, Argentina

Mathias Mølbak Ingholt, United Kingdom

Cláudia Jardim Santos, Portugal

Torsten John, Germany

Ania Kawiecki, Spain

Hadi Kazemi-Arpanahi, Iran

Michiko Kikuchi, Japan

Gretchen King, Lebanon

Christina Koscher-Kien, Austria

Didier Koumavi Ekouevi, Togo

Emanuel Krebs, Canada

Nigel Laing, Australia

Barbara Löfflad-Bürkin, Switzerland

Henrique Lopes, Portugal

Nicola Love, United Kingdom

Sri Novita Lubis, Indonesia

Jakub Marcinkowski, Poland

Olivier Marcy, France

Jose M. Martin-Moreno, Spain

Sebastià Mas Alòs, Spain

Landry Ndriko Mayigane, Switzerland

Kathleen Mcgee, Switzerland

Charlotte Meixner, Germany

Alfredo Mena Lora, United States

Alexey Mikhaylov, Russia

Anjali Modi, India

James Mooney, United States

Marlene Muehlmann, Germany

Sulaiman Muhammad Musa, Nigeria

Sianga Mutola, Germany

Ruth Namazzi, Uganda

Ifeoma Nduka, Nigeria

Natalia Giraldo Noack, Germany

Willia Novita Eka Rini, Indonesia

Takuya Onoda, Germany

Virgile Pace, Madagascar

Marcelo Benedeti Palermo, Brazil

Martina Paric, Netherlands

David Pérez-Jorge, Spain

Julia Pescarini, United Kingdom

Laoraksawong Pokkamol, Thailand

Anung Ahadi Pradana, Indonesia

Adriyan Pramono, Indonesia

Hugo Quezada Pinedo, United States

Peter Francis Raguindin, Switzerland

Susan Rasmussen, United Kingdom

Birgit Reime, Germany

Ana Isabel Ribeiro, Portugal

Pablo Rodríguez-Feria, Netherlands

Anna Romanova, Switzerland

Daniel Saldanha Resendes, Portugal

Blanca Salinas-Roca, Spain

Catarina Samorinha, Portugal

Robert Adamu Shey, Cameroon

Rahul Shidhaye, India

Iryna Shubets, Netherlands

Koffi Mawugbe Siliadin, Norway

Pimpan Silpasuwan, Thailand

Flávio Silva, Brazil

Racheal M. Smetana, United States

Penpatra Sripaiboonkij, Ireland

Mindaugas Stankunas, Lithuania

Lara Stucki, Spain

Marwan Suluiman, Sudan

Dewi Susanna, Indonesia

Osama Tanous, United States

Abhinand Thaivalappil, Canada

Clémence Thébaut, France

Deborah Thomson, United States

Philipp Trein, Switzerland

Eni Tresa, Albania

Justine Guguneni Tuolong, Ghana

Ndadilnasiya Endie Waziri, Nigeria

Rona Weeras, Australia

Frank Wieber, Switzerland

Tenglong Yan, China

Nicholas Yell, United States

Konstantinos Zacharis, Greece

Shibo Zhang, China

